# Study on Emission Characteristics and Emission Reduction Effect for Construction Machinery under Actual Operating Conditions Using a Portable Emission Measurement System (Pems)

**DOI:** 10.3390/ijerph19159546

**Published:** 2022-08-03

**Authors:** Junhui Chen, Yuan Li, Zhongwei Meng, Xiaoqiong Feng, Junjie Wang, Honghui Zhou, Junjie Li, Jiacheng Shi, Qiang Chen, Hongle Shi, Shuxiao Wang

**Affiliations:** 1 School of Environment, Tsinghua University, Beijing 100084, China; xxcdcjh@163.com (J.C.); shxwang@tsinghua.edu.cn (S.W.); 2 Sichuan Academy of Environmental Sciences, Chengdu 620041, China; yoileeshine@outlook.com (Y.L.); m.s.wangjunjie@foxmail.com (J.W.); xsnjzljj@hotmail.com (J.L.); sjiach@163.com (J.S.); chengqiang1999@126.com (Q.C.); shl101@163.com (H.S.); 3 Sichuan Environmental Protection Key Laboratory of Moving Source Pollution and Control, Chengdu 620041, China; 4 School of Energy and Power Engineering, Xihua University, Chengdu 610039, China; mengzw@mail.xhu.edu.cn; 5 Sichuan Province Environmental Protection Technology Engineering Co., Ltd., Chengdu 620041, China; f_xiaoqiong@126.com

**Keywords:** construction machinery, PEMS, emission reduction techniques, emission reduction effect

## Abstract

With the acceleration of urban construction, the pollutant emission of non-road mobile machinery such as construction machinery is becoming more and more prominent. In this paper, a portable emissions measurement system (PEMS) tested the emissions of eight different types of construction machinery under actual operating conditions and was used for idling, walking, and working under the different emission reduction techniques. The results showed that the pollutant emission of construction machinery is affected by the pollutant contribution of working conditions. According to different emission reduction techniques, the diesel oxidation catalyst (DOC) can reduce carbon monoxide (CO) by 41.6–94.8% and hydrocarbon (HC) by 92.7–95.1%, catalytic diesel particulate filter (CDPF) can reduce particulate matter (PM) by 87.1–99.5%, and selective catalytic reduction (SCR) using urea as a reducing agent can reduce nitrogen oxides (NO_x_) by 60.3% to 80.5%. Copper-based SCR is better than vanadium-based SCR in NO_x_ reduction. In addition, the study found that when the enhanced 3DOC + CDPF emission reduction technique is used on forklifts, DOC has a “low-temperature saturation effect”, which will reduce the emission reduction effect of CO and THC. The use of Burner + DOC + CDPF emission reduction techniques and fuel injection heating process will increase CO’s emission factors by 3.2–3.5 and 4.4–6.7 times compared with the actual operating conditions.

## 1. Introduction

With the rapid development of society, the demand for construction machinery in urban construction is increasing [1]. The construction machinery emissions are also becoming more serious [2]. Since construction machinery uses fuel, mainly diesel, its NO_x_ and particulate matter (PM) emissions are usually higher than those of other fuels such as gasoline [3]. Emissions of vehicle pollutants can harm human health and the environment [4]. According to statistics, in 2016, 2850 tons of nitrogen oxide emissions from non-road mobile machinery in the construction sector in London accounted for 7% of the total nitrogen oxide emissions in society [5]. Since the emission standards of construction machinery lag behind the emission standards of on-road vehicles [6], the emission limit requirements of construction machinery are relatively low. The NO_x_ emission limit of the sixth-stage vehicle test for heavy-duty diesel vehicles is 690 mg/kWh [7], and the fourth-stage NO_x_ emission limit for non-road mobile machinery diesel engines is 0.67–3.5 g/kWh in Beijing [8]. Controlling the pollutant emission of construction machinery will be a significant project to reduce the emission of mobile sources in the future [7,8].

Since September 2017, the use of Portable Emission Measurement Systems (PEMS) for testing real-world driving emissions (RDE) inappropriate road circuits has been a mandatory element of the certification of new vehicle types for passenger vehicles worldwide [9]. Since then, the non-road China-IV upgrade and the PEMS (Portable Emissions Measurement System) method for non-road machinery emissions testing have been on the agenda. In order to clarify the emission situation of non-road mobile sources and to carry out pollution prevention and control of non-road mobile machinery, many researchers have conducted PEMS tests for non-road mobile sources. Zhang et al. [10] studied the PM2.5 emission characteristics of construction machinery under different operating conditions using a portable particulate matter dilution sampling system, but no study was conducted for gaseous pollutant emission characteristics. Abolhasani et al. [11] studied the emission characteristics of major gaseous pollutants and particulate matter from excavators using a portable emission measurement system, but no comparative experiments were conducted for different machine types. Based on the PEMS system, Ye et al. [12,13,14] did not conduct emission reduction test studies of different abatement technologies, although they studied the pollutant emission characteristics of different machinery types. Ge et al. [15,16,17] showed that the technical route of using DOC, DPF, SCR single, or a combination of multiple after-treatment devices on the engine bench could effectively reduce the pollutant emissions of diesel engines. However, there is a lack of emission reduction experiments under actual operating conditions. Through the research of the above literature, this study obtained the theoretical issues such as the design of experimental conditions, the selection of experimental machinery, and the setting of abatement technologies from it, which provided methodological and theoretical support for this study. This study makes up for the pollutant emission characteristics of different machinery types, and most importantly, it investigates the emission reduction benefits by combining different emission reduction technologies, which can provide more data support for the emission reduction of construction machinery and policy formulation, and is of great significance to solve the pollution prevention problems of construction machinery.

The primary purpose of this study is to use the PEMS system to test and study the pollutant emission characteristics of different construction machinery under actual operating conditions and obtain the advantages or disadvantages of different emission reduction techniques. It is expected to provide data support for establishing a local non-road mobile emission source emission inventory and formulating emission reduction measures for construction machinery.

## 2. Methodology

### 2.1. Experimental Equipment

**Figure 1:**
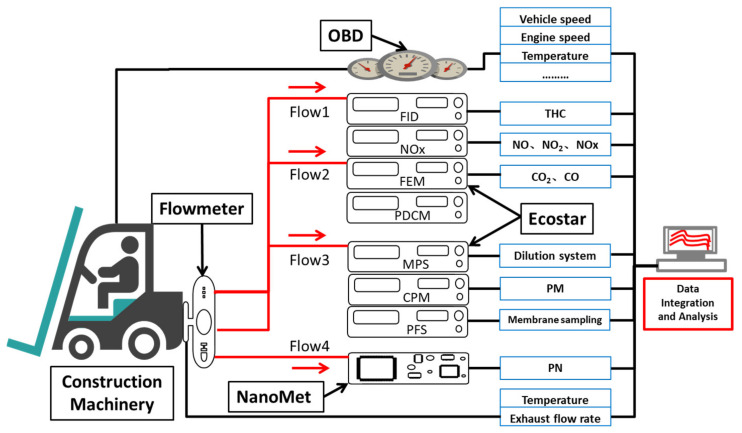
Schematic diagram of the structure of the portable exhaust emission testing system (PEMS). The PEMS structure schematic shown in the picture was built for this study.

In this study, the PEMS (Portable Emissions Measurement System) was used to collect real-time data on the pollutant emissions of selected construction machinery under actual operating conditions. The PEMS test system was used to study the existing examples of motor vehicle pollutant emissions [18,19,20]. As shown in Figure 1, The portable exhaust gas measurement system (PEMS) used in this study is the SEMTECH-ECOSTAR equipment of Sensors Company in the United States. The system is mainly composed of ① Heating the sampling flow tube; ② Mechanical operating parameters (speed, load, exhaust temperature, exhaust flow, etc.), collector OBD; ③ FID (hydrogen ion flame method to measure THC), NO_x_ (non-dispersive ultraviolet light method to measure NO, NO_2_), FEM (non-dispersive infrared light method to measure CO, CO_2_) and other gas pollutant analysis modules; ④ MPS (Sampling Dilution System), CPM (PM), PFS (Membrane Sampling System) and other particulate matter sampling and analysis modules. The NanoMet equipment was also used for the compliance test of construction machinery off-road four-stage pollution limit, which can realize the collection of particulate matter number (PN) of exhaust gas [21].

### 2.2. Selection of Experimental Machinery and Emission Reduction Techniques

According to the China Mobile Source Environmental Management Annual Report (2021) results, excavators, loaders, and forklifts are the top three types of construction machinery with pollutant emissions. The pollutant emissions of the three types of machinery accounted for more than 90% of the construction machinery. This study selected three types of machinery: Excavators, loaders, and forklifts. As shown in Table 1, the selected machinery meets the emission stages from China-I to China-IV, and the rated power range covers 35.4–298 kW. Based on the research of other scholars, this study proposes to use different emission reduction techniques for different types of machinery to verify their emission reduction effect in the actual operating conditions of machinery.

### 2.3. Design of Experimental Conditions

Due to the complex changes in operating conditions of construction machinery in the process of actual operation, in the purchase of exhaust emission characteristics of different machinery types and different operating conditions, the experimental cycle designed in this study refers to the emission characteristics study of an actual operating process of non-road mobile machinery by Qu et al. [10,14,22]. Under the three conditions of idling, walking, and working, the same is that all mechanical conditions are walking before and after the preset position, and no other operations are performed. The difference is that the idling condition of loaders and forklifts is the lowest speed operation at which the machinery remains stationary, and the engine is in the state of the combustion engine. The idling conditions of the excavator include idling operation in different gears (10th, 8th, 6th, 4th, and 2nd), the excavator test cycle time is 2700 s, and the loader and forklift test cycle time is 3600 s.

### 2.4. Experiment Implementation Process

The experiments conducted in this study all required the completion of five processes: starting the experimental preheating equipment, calibrating the experimental equipment, starting the preheating construction machinery, completing the experimental data collection, and processing the experimental data. The post-processing systems used were all newly installed for the abatement experiments conducted on construction machinery. During the experimental data collection process, exhaust gas samples were collected from each construction machine before and after the post-treatment system installation. The advantages and disadvantages of different emission reduction techniques were approved based on the differences in pollutant emissions before and after the use of emission reduction techniques.

### 2.5. Data Analysis of Experimental Results

#### 2.5.1. Quality Control and Processing of Experimental Data

The accuracy of the experimental sampling equipment was ensured. Before each experimental test, the Ecostar equipment used standard gas to calibrate each module and N_2_ 99.9999% standard gas to zero-calibrated the gaseous pollutant collection module. The gaseous pollutant acquisition module was calibrated using standard gas with CO_2_ 14%, CO 5000 ppm, NO 2000 ppm, and propane 303 ppm as the background gas of N_2_. A separate standard calibration of NO_2_ was carried out with the standard gas of NO_2_ 300 ppm, and the background gas was air. After the experiment, the standard calibration was also carried out with the above gases. It was verified that the drift of each module during the experiment achieved the standard requirements. The emission rate of pollutant mass per second under actual operating conditions was obtained by using the experimental data through Formulas (1)–(5) [7,8].

(1) $$C_{wet}=k_{w}\times C_{dry}$$

where $C_{wet}$ is the contaminant wet base concentration, the unit is ppm, or volume percentage; $C_{dry}$ is the dry concentration of pollutants, in ppm, or volume percentage; $k_{w}$ is the dry and wet basis correction factor.

(2) $$k_{w}=\left(\frac{1}{1+\alpha \times 0.005\left(C_{CO_{2}}+C_{CO}\right)}-\frac{1.608\times H_{a}}{1000+\left(1.608\times H_{a}\right)}\right)\times 1.000$$

where $H_{a}$ is the absolute humidity of the intake air, in gH_2_O/kg dry air; $C_{CO_{2}}$ is the dry basis CO_2_ concentration, %; $C_{CO}$ is the dry basis CO concentration, %; α is the hydrogen molar ratio.

(3) $$R_{NO_{xi}}=\frac{0.001587\times NO_{xconc}\times G_{exh}}{3600}$$

(4) $$R_{CO_{i}}=\frac{0.000966\times CO_{conc}\times G_{exh}}{3600}$$

(5) $$R_{THC_{i}}=\frac{0.000479\times HC_{conc}\times G_{exh}}{3600}$$

where $R_{NO_{xi}},\;R_{CO_{i}},\;R_{THC_{i}}$ are the instantaneous emissions of each gaseous pollutant, g/s; $NO_{xconc},\;CO_{conc},\;HC_{conc}$ (hydrocarbons are expressed in C1 equivalents (total carbon), with C1 representing the equivalent hydrocarbon of 1 carbon) are the instantaneous wet base concentration of each gaseous pollutant in the original exhaust gas, ppm; $G_{exh}$ is the instantaneous exhaust flow, kg/h.

#### 2.5.2. Analysis of Experimental Data

The main contents of the experimental data are ① calculation of pollutant emissions and emission factors of machinery; ② calculation of emission reduction effects of different machinery types using different emission reduction techniques under actual operating conditions. The emission calculation of relevant data was completed based on the pollutant emission data obtained from the actual operating conditions in the previous chapters.

Since most of the tested machinery in this study is old machinery, the collection of mechanical engine operating parameters cannot be completed by the equipment with its own OBD, and this study used the carbon balance method [23] to calculate the fuel consumption of machinery, which is calculated in Equation (6), and the emission factor of mechanical pollutants based on fuel consumption was obtained by combining the emissions of relevant pollutants.

(6) $$DCR_{i}=\frac{\left(0.273*R_{CO_{2i}}+0.429*R_{COi}+0.866*R_{THCi}\right)}{CWF_{F}*\rho }$$

where $DCR_{i}$ is the diesel consumption rate, L/s; $R_{CO_{2i}},\;R_{COi}$ and $R_{THCi}$ are the mass emission rates of CO_2_, CO and HC, respectively, g/s; 0.273, 0.429 and 0.866 are the carbon content of CO_2_, CO and HC, respectively; *CWF_F_* is the carbon-to-mass ratio of diesel, the reference value in this study is 0.866, and *ρ* is the density of diesel under standard conditions, which is 0.849 kg/L.

(7) $${\mathrm{DEF}}_{i}=\frac{\sum_{1}^{n}R_{i}}{\sum_{1}^{n}{\mathrm{DCR}}_{i}*\rho }$$

where ${\mathrm{DEF}}_{i}$ is the emission factor of pollutants based on fuel consumption, g/kg; ${\mathrm{DCR}}_{i}$ is the diesel consumption rate per second, L/s; $R_{i}$ is the mass emission rate of pollutants, g/s.

Since the exhaust emissions of construction machinery are greatly influenced by the actual operating conditions of the machinery, this study combined field research and literature research to obtain the working time share of machinery: loader and forklift idling 10%, walking 40%, and working 50%, and excavator idling 11%, walking 15%, and working 74%. According to the different operating condition percentages [18], the comprehensive emission factor based on fuel consumption was estimated according to Equation (8).

(8) $$CEF_{i}=\overset{n}{\underset{j=3}{\sum }}DEF_{i,j}\times T_{j}$$

where $CEF_{i}$ is the comprehensive emission factor of pollutants, i refers to the type of pollutant; $DEF_{i,j}$ is the average emission factor (g/kg) of the pollutant in the operating condition, j refers to mechanical conditions, i.e., idling, walking, working; n is the number of test condition types, which is 3; $T_{j}$ refers to the proportion of time that the machine completes an experimental cycle condition j. The advantage of using the estimation method is that it is more comparable to analyzing the same machinery type emission characteristics [2]. The estimation method also has the disadvantage of not fully characterizing the actual working condition emissions of each piece of machinery because the proportion of working conditions of each piece of construction machinery is inconsistent [24,25].

To verify the emission reduction effect of the pollutants of construction machinery using different emission reduction techniques, the pollutant emission data before and after the machinery using emission reduction techniques were collected, and the comprehensive emission factors of each pollutant were obtained by the above-mentioned emission factor calculation method, and this study will be based on the results of the pollutant emission factors before and after the machinery using emission reduction techniques, and the proportion of pollutant emission reduction was calculated according to Formula (9) to characterized the emission reduction effect of different emission reduction techniques by the proportion of emission reduction.

(9) $${\mathrm{ERR}}_{i,j}=\frac{{\mathrm{CEF}}_{\mathrm{fi},j}-{\mathrm{CEF}}_{\mathrm{ai},j}}{{\mathrm{CEF}}_{\mathrm{fi}},j}\times 100\%$$

where ${\mathrm{ERR}}_{i,j}$ is the emission reduction ratio of pollutants in operating conditions, %; ${\mathrm{CEF}}_{\mathrm{fi},j}$ is the pollutant emission factor before using the emission reduction techniques, g/kg; ${\mathrm{CEF}}_{\mathrm{ai},j}$ is the pollutant emission factor before using the emission reduction technique, g/kg; j refers to mechanical operating conditions, i.e., idling, walking, working.

## 3. Results and Discussion

**Figure 2:**
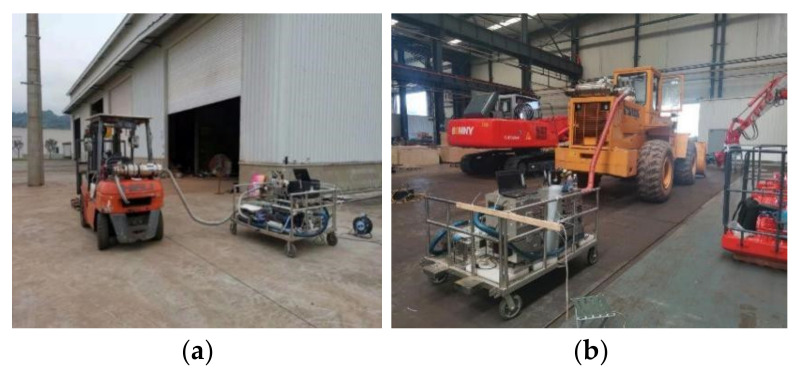

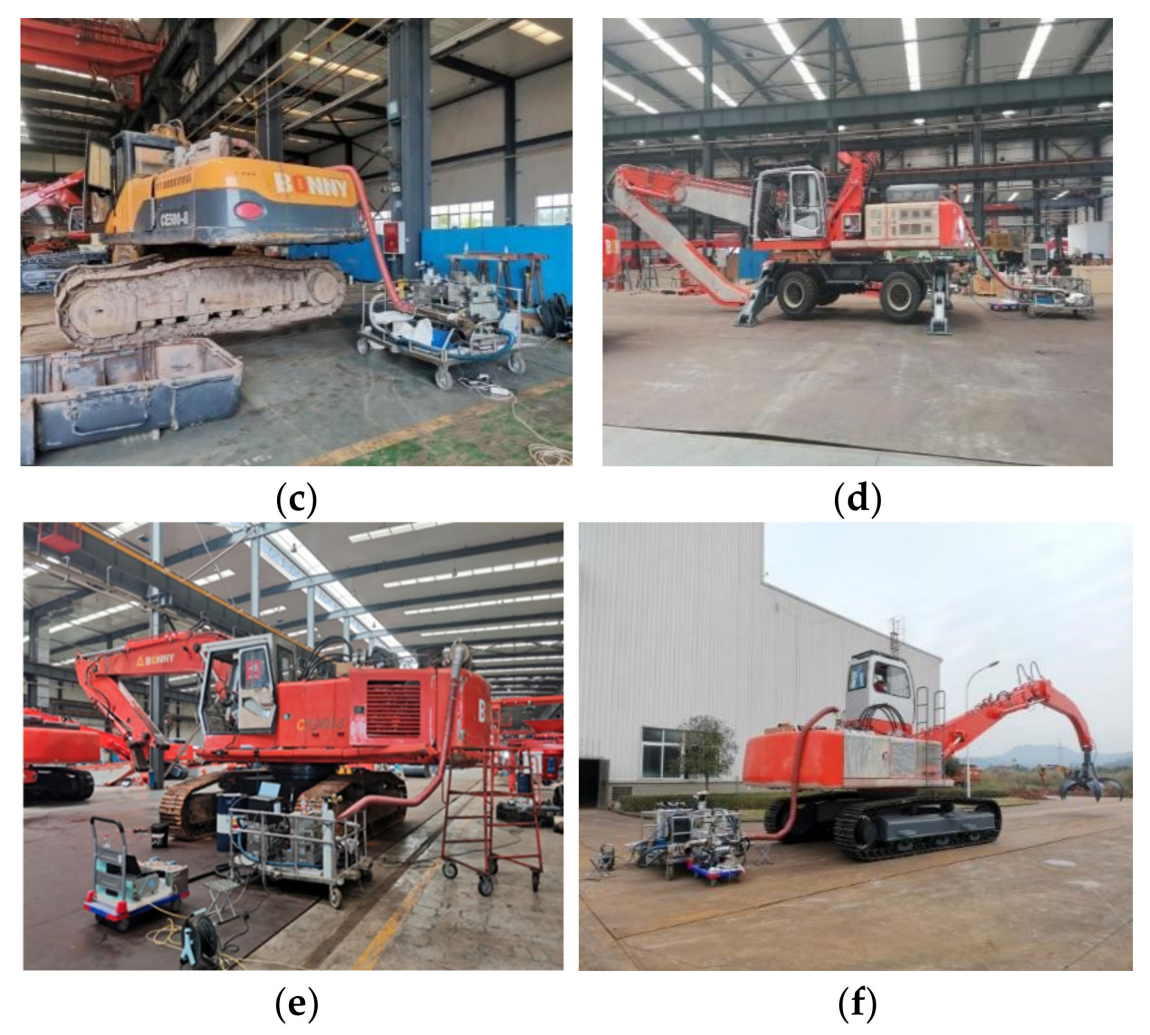
Part of the field experiment diagram of measuring machinery. (**a**) Description of Forklift walking condition test; (**b**) Description of loader idling condition test; (**c**) Description of excavator idling condition test before installation of the treatment unit; (**d**) Description of excavator walking condition test; (**e**) Description of excavator idling condition test after installation of the treatment unit; (**f**) Description of excavator working condition test. The pictures were actually taken during the field tests of this study.

The discussion and analysis of the experimental results in this study mainly include: (1) the pollutant emission characteristics of different types of machinery; (2) the emission reduction characteristics and proportions of different types of machinery using different emission reduction techniques. The experimental site is shown in Figure 2.

### 3.1. Emission Characteristics of Different Types of Machinery

**Figure 3:**
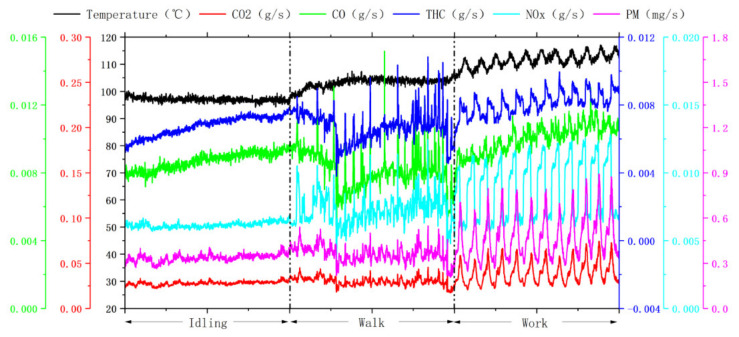
Instantaneous emission mass rate and temperature of pollutants under three operating conditions of forklift truck (engine power 35.4 kW).

**Figure 4:**
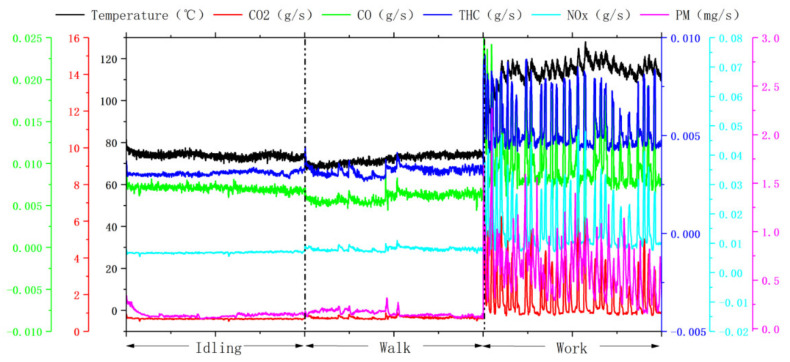
Instantaneous emission mass rate and temperature of pollutants under three operating conditions of the loader (engine power 162 kW). Due to the limitation of the experimental conditions, the walking conditions of the loader were sampled under the low-speed walking conditions.

**Figure 5:**
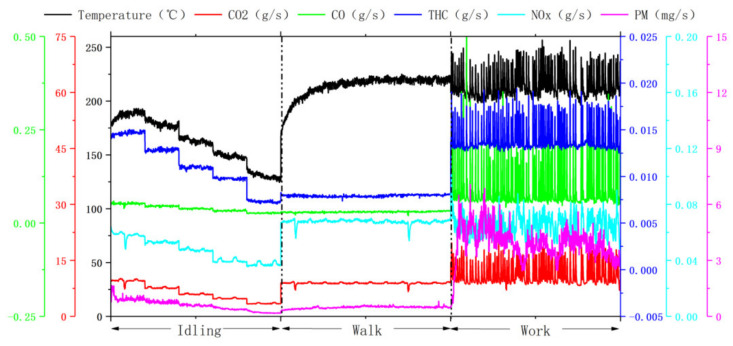
Instantaneous emission mass rate and temperature of pollutants under three operating conditions of excavator (engine power 298 kW).

This study tested the pollutant emission rates of different machinery under different operating conditions. Figure 3, Figure 4 and Figure 5 show that the actual operating conditions influence the pollutant emission of construction machinery, and the emission of working conditions is much higher than that of idling and walking conditions. From the viewpoint of machinery types, the pollutant emissions of forklift working conditions were 1.2–2.7 and 1.2–2.3 times that of idling and walking conditions. The pollutant emissions of loader working conditions were 2.8–4.0 and 1.8–2.2 times of idling and walking conditions, respectively, and the pollutant emissions of excavator working conditions were 2.3–8.5 and 2.2–6.7 times of idling and walking condition respectively. Due to frequent operation, construction machinery in operation results in changes in engine speed and torque, bringing about drastic changes in combustion chamber combustion conditions, resulting in a rapid increase in pollutant emissions.

Table 2 compares the measured factors of pollutants in this study and other researchers. It can be seen from Table 2 that the pollutant emission factors obtained from different studies are quite different. This is mainly due to the lack of unified testing methods and specifications. Additional research, testing equipment, and testing principles are also different, and the selection of test objects is also different. For example, for NO, this study and Fu [24] used a non-dispersive ultraviolet (NDUV) sensor, and Pang [25] and Frey [26] used a chemical sensor. For PM, the diffusion charge method was used in this study, the laser scattering method was used for Fu and Frey and Pang used the filter weighing method. In addition, there are many reasons for the different emissions of different mechanical pollutants [14], including different mechanical emission stages, mechanical power, mechanical work intensity, and driver operating habits, which will make the results of each test experiment inconsistent. Comparing the results of this study with different types of machinery, the emission factors of various pollutants of the forklift are 2–20 times that of the loaders and excavators tested in the experiment. The pollution emissions are even worse, which is closely related to the emission stage of China-I but also related to factors such as differences in operating conditions and engine maintenance levels. Due to their higher exhaust temperatures, excavators and loaders have higher NO_x_ emission factors than forklifts. From the emission stage, the China-III, China-II stage machinery emission factors of various pollutants are significantly smaller than the China-I stage. On the one hand, the reason may be due to the machinery activities, and operational content caused. On the other hand, it may be due to the non-road machinery emission standards in recent years caused by the tightening.

**Figure 6:**
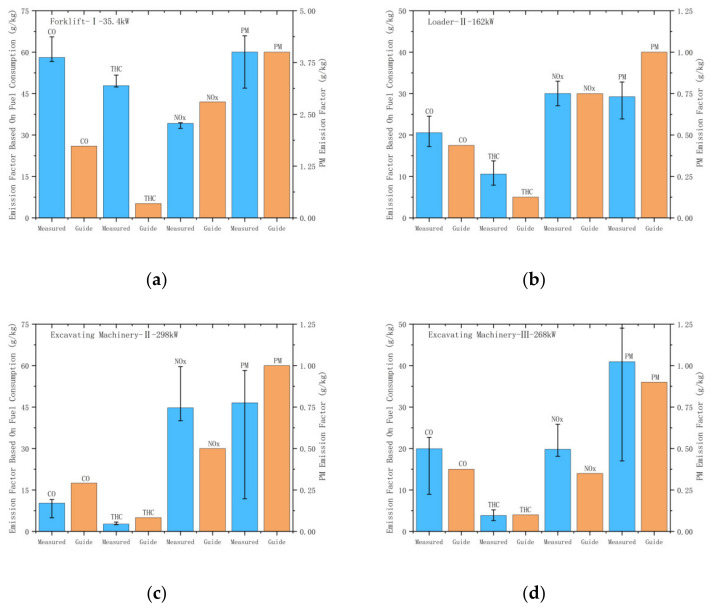
Comparison between measured factors and guide factors of construction machinery based on fuel consumption. (**a**) Comparison between the measured factor and guide factor of China-I forklift; (**b**) Comparison between the measured factor and guide factor of China-II loader; (**c**) Comparison between the measured factor and guide factor of China-II excavator; (**d**) Comparison between the measured factor and guide factor of China-III excavator.

Based on the measured pollutant emission results of construction machinery and the typical time proportion of each operating condition investigated [25], the comprehensive emission factor of each pollutant based on fuel consumption was obtained in this study. Since fuel consumption is relatively easy to obtain [2], pollutant emission factors based on fuel consumption are widely used to establish emission inventories for non-road mobile sources [24,25,26]. As shown in Figure 6, compared with the recommended values in the literature [27], this study is measured, and comprehensive emission factors are quite different from those in the literature. It can be seen that the calculation of emission inventories using the guideline-recommended factors may underestimate the emission of some pollutants, which also highlights the importance of conducting actual emission tests on non-road machinery under operating conditions for constructing relatively accurate emission factors. Since the number of tested machines in this study is relatively small, it has certain limitations for analyzing emission characteristics. Therefore, more sample tests are essential in analyzing emission characteristics and constructing an emission factor library.

### 3.2. Emission Reduction Characteristics of Different Emission Reduction Techniques

In this study, the emission factors of different pollutants based on fuel consumption were calculated by collecting the pollutant emission data of construction machinery before and after using emission reduction techniques. The pollutant emission reduction ratio was obtained using the difference of emission factors before and after, as shown in Table 3. From the study results, various emission reduction techniques can make construction machinery pollutants reduce 12.8–99.5% under actual operating conditions, and from different devices, DOC, it can make CO reduce 41.6–94.8%, make THC reduce 92.795.1%, and for CDPF, it can reduce PM by 87.2–99.5%, and for SCR, it can reduce NO_x_ by 60.3–80.5%. The emission reduction ratio of copper-based SCR obtained in this project is better than that of vanadium-based SCR performance, which is consistent with the results of Wang et al. [28], who showed that the overall performance of copper-based molecular sieve catalysts is better than that of vanadium-based catalysts. This shows that adding DOC, CDPF, and SCR at the back end of construction machinery exhaust can effectively reduce pollutant emissions.

It is crucial to mention that this study can reduce CO by 12.8% and PM by 72% using Eco Clean Fuel + lubricant additive without adding any post-treatment device to machinery No. 6. The reason for this is that Eco Clean Fuel is a clean diesel produced by adding Eco Booster to regular motor diesel. Eco Clean Diesel has a suitable oxygen content and higher cetane number than regular motor diesel. Increasing the oxygen content of diesel fuel reduces the unevenness of oxygen concentration in the cylinder, relieves the lack of oxygen in the local combustion area in the cylinder, and makes the fuel burn more fully; the increase of cetane number can improve the ignition of diesel fuel, shorten the stall period, make the engine work smoothly and make the fuel burn entirely in the combustion chamber. Thus, it can be seen that the improvement and optimization of oil quality is another effective measure to reduce mechanical pollutants.

Combined with the results of other researchers, using single or combined DOC, DPF, and SCR post-treatment systems can effectively reduce the emission of pollutants. However, the same post-treatment system has some differences in the reduction effect of pollutants, the treatment efficiency of DOC for CO in this study is lower than the results of other researchers, and the treatment efficiency of DPF for PM is slightly higher than the results of other researchers, the reason for this is that the type and ratio of catalyst used to affect the treatment efficiency of pollutants [34].

Given the outstanding performance of forklift pollutants, this study focuses on analyzing the emission reduction characteristics of two different emission reduction techniques on forklift trucks. The differences between the two emission reduction techniques are: Forklift 1 used three DOCs; Forklift 2 installed a set of exhaust temperature control systems at the front end while using one DOC. The system used its fuel injection device to inject the reserve diesel into the front end of the burner and used the exhaust gas temperature to ignite the injected diesel to increase the exhaust gas temperature. From the perspective of emission reduction ratio, the main difference between the two was reflected in the emission reduction of CO and THC. Using the burner + DOC + CDPF emission techniques is more conducive to the emission reduction of CO and THC pollutants. The reason is that the fuel injection of the burner increases so that the temperature entering the DOC is more suitable for the action temperature of the coating catalyst, which is more conducive to the emission reduction of both.

**Figure 7:**
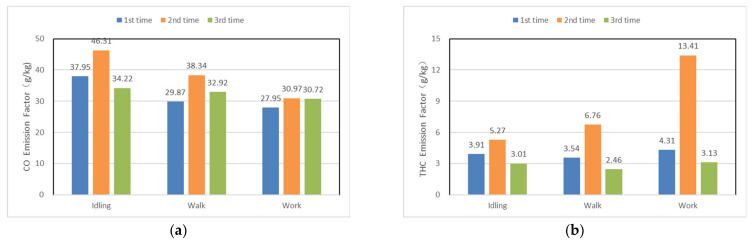
Emission factors of CO and THC based on fuel consumption for three consecutive test cycles of forklift trucks using the enhanced 3DOC + CDPF techniques. (**a**) Description of CO emission factor for forklift for 3 consecutive test cycles; (**b**) Description of THC emission factor for forklift for 3 consecutive test cycles.

**Figure 8:**
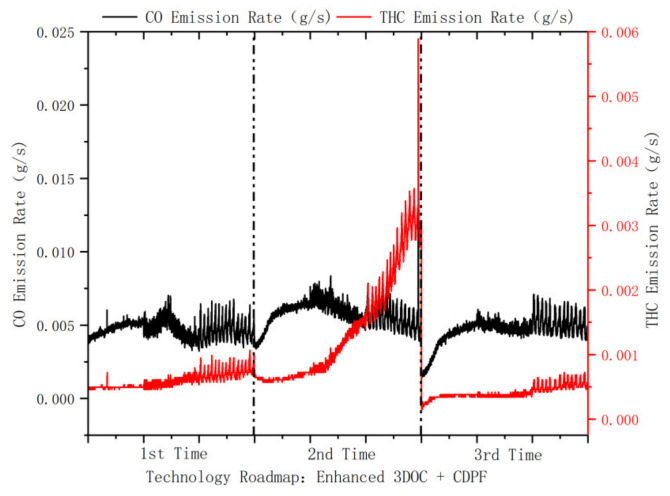
CO and THC emission rates of forklifts using enhanced 3DOC + CDPF technology.

Figure 7 shows the CO and THC emission factors of forklift 2 for three consecutive test cycles when using the enhanced 3DOC + CDPF emission reduction techniques. All three cycles were carried out according to the designed experimental conditions (idling, walking, and working), with the difference that the forklift had a high load condition operation after the second cycle, followed by the third experimental cycle. The CO and THC emission factors of the 2nd test cycle were higher than those of the first and third cycles. From the CO and THC emission rate graphs (Figure 8), it can be seen that the THC emission rate showed a rapid increase after the idling condition of the second experimental cycle, which was analyzed because the original engine exhaust temperature was low (90–120 °C) during the experimental test. The DOC was able to capture and regenerate CO and THC during the extended time low-temperature conditions. In the process of regeneration, the “low-temperature saturation effect” occurred, which led to the reduction of the effect of DOC on CO and THC emission reduction. Compared with the second cycle, the THC emission rate of the third experimental cycle was significantly reduced because the forklift was operated under high load conditions after the second test cycle to regenerate the pollutants adsorbed by the DOC due to the “low-temperature saturation effect” at high temperature, and the high-temperature regeneration process could quickly remove the pollutants adsorbed by the DOC. After removing the pollutants adsorbed on top of DOC, in the third test cycle, DOC has better adsorption and capture effect on CO and THC. This shows that the “low-temperature saturation effect” of DOC is more affected by THC.

**Figure 9:**
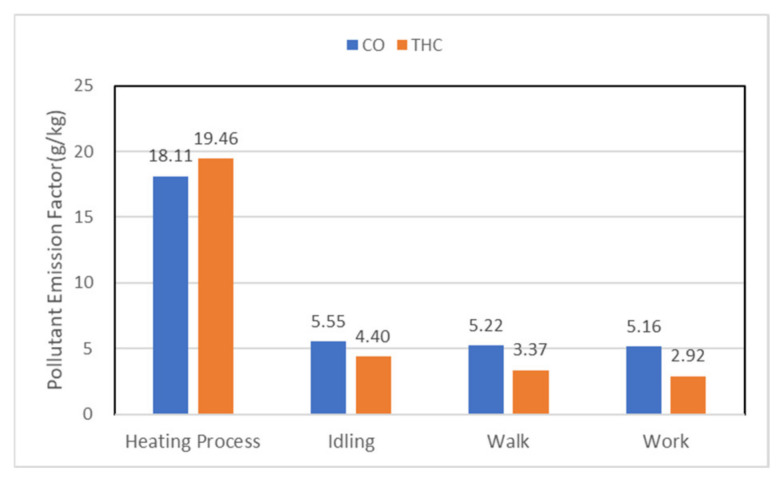
CO and THC emission diagram of forklift using burner + DOC + CDPF emission reduction techniques.

As shown in Figure 9, in fuel injection heating, the CO emission factor was 3.2–3.5 times that of the actual operating condition, and the THC was 4.4–6.7 times. Because the system used the burner to inject reserve diesel into the exhaust pipe to increase the exhaust temperature. Because the temperature of DOC did not reach the appropriate temperature, it cannot effectively deal with the incomplete combustion of the injected fuel, resulting in a sudden increase in CO and THC. After the exhaust gas emission temperature had been stabilized and reached the suitable temperature of DOC, the burner dynamically adjusted the fuel injection valve so that the DOC ran stably, and the treatment effect of CO and THC was stable at the moment.

## 4. Conclusions

Operating conditions significantly influence pollutant emissions from construction machinery. In particular, the contribution of pollutants from operating conditions is significant. Forklift trucks in China-I are highlighted among the tested machines. Different emission reduction techniques can make construction machinery in actual operating conditions reduce emissions by 12.8–99.5%, copper-based SCR for NO_x_ emission reduction effect is better than the vanadium-based SCR. With appropriate oxygen content, the higher cetane number of environmentally friendly clean diesel fuel + lubricant additives can reduce CO and PM emissions without increasing the construction machinery after-treatment device. There is a “low-temperature saturation effect” with DOC in the low exhaust temperature conditions for a long time. This effect will lead to reduced emission reduction techniques for CO and THC emission reduction effect. The emission reduction technology of Burner + DOC is more conducive to CO and THC emission reduction. Compared to the actual operating conditions, the injection process of warming up will make CO and THC emissions rise. The measurement results from this study show that the improvement of emission standards, fuel quality, and emission after-treatment modification are effective measures to make construction machinery reduce emissions under actual operating conditions. This study supplements the emission reduction effect test of emission reduction technologies under actual operating conditions of construction machinery. This study can provide a reference for future studies on emission reduction of post-treatment systems such as DOC, DPF, and SCR under actual operating conditions. In the future, this proposed method can offer a reference for studying emission reduction of DOC, DPF, SCR, and other after-treatment systems under actual operating conditions. In addition, it provides the necessary support for establishing a construction machinery emission factor library and constructing an emission inventory study and provides technical guidance for developing construction machinery pollution prevention and control policies and emission reduction assessment.

## Figures and Tables

**Table 1:** Basic information on testing construction machinery.

No.	Mechanical Type	Machine Brand	Engine Brand	Engine Model	Engine Rated Power (kw)	Rated Speed (r/min)	Engine Cylinders	Engine Displacement (L)	Emission Stage	Emission Reduction Techniques
**1**	Forklift	HELI	ISUZU	ISUZU C240PKJ	35.4	2500	4	2.4	China-I	Enhanced model 3DOC + CDPF
**2**	Forklift	HELI	ISUZU	ISUZU C240PKJ	35.4	2500	4	2.4	China-I	Burner + DOC + CDPF
**3**	Loader	LIUGONG	WEICHAI	WD625G 220	162	2100	6	9.7	China-II	DOC + CDPF + Copper-based molecular sieves SCR + ASC
**4**	Excavator	YUCHAI	CUMMINS	M22	298	2100	6	10.8	China-II	DOC + CDPF + Vanadium based SCR + ASC
**5**	Excavator	BONNY	CUMMINS	QSM22	268	2100	6	10.8	China-III	DOC + CDPF + Vanadium based SCR + ASC
**6**	Excavator	BONNY	CUMMINS	QSB7	169	2050	6	6.7	China-III	Clean fuel + lubricant additives
**7**	Excavator	BONNY	CUMMINS	QSB7	169	2050	6	6.7	China-III	DOC + CDPF + Copper-based molecular sieves SCR + ASC
**8**	Grabber	BONNY	CUMMINS	Match only	180	2000	6	6.7	China-IV	DOC + CDPF + Copper-based molecular sieves SCR + ASC

The reducing agents for SCR systems in the table are all urea.

**Table 2:** Comparison of emission factor results for different types of construction machinery (g/kg).

Mechanical Type	Power Range	Emission Standards	CO			HC			NO			PM			Remark
			Idling	Walking	Working	Idling	Walking	Working	Idling	Walking	Working	Idling	Walking	Working	
Forklift	<37	China-I	70.8	60.9	59.9	53.6	49.1	48.6	31.2	37.1	39	3.1	3.5	4.2	This Study (Table 1 No. 1)
Forklift	<37	China-I	60.3	55.1	53.3	49.7	45.7	46.4	9.4	10	10.7	3.2	3.9	4.6	This Study (Table 1 No. 2)
Forklift	37 ≤ *p* < 75	China-0	43.7	9.6	26.3	14.2	12	10.9	8.8	10.1	14.9	4.9	1.3	8.1	Pang [25]
Forklift	75 ≤ *p* < 130	China-I	27.3	23.7	16.7	6.7	6.6	3.6	15.9	20.7	13.7	1.7	15.3	3.4	Pang [25]
Loader	*p* ≥ 130	China-II	21.3	24.6	17.2	13.8	13.1	7.9	25.6	25.9	23.8	0.6	0.7	0.8	This Study (Table 1 No. 3)
Loader	*p* ≥ 130	China-II	43.5	32.3	23.1	3.5	1.8	0.4	3.2	9	7.7	5.4	4.7	1.9	Pang [25]
Loader	/	/	21.4			1.2			60.1			4			Xia [2]
Loader	/	/	17.5			7.6			83.4			1.5			Frey [26]
Loader	/	/	11			6			42.6			0.3			Fu [24]
Excavator	*p* ≥ 130	China-II	8.8	4.9	11.5	3.4	2.4	2.6	52.9	52	36.1	0.3	0.2	1	This Study (Table 1 No. 4)
Excavator	*p* ≥ 130	China-II	14.5	15.9	5.9	4.3	2.3	2.4	20.1	32.8	22.4	1.1	3.1	2.5	Pang [25]
Excavator	*p* ≥ 130	China-III	16.6	9	22.7	5.2	2.6	3.9	21.6	21.5	15.4	0.5	0.4	1.2	This Study (Table 1 No. 5)
Excavator	37 ≤ *p* < 75	China-III	28.9	12.3	11.3	9.6	0.9	0.8	19.9	49	45.3	3.6	2.3	3.9	Pang [25]
Excavator	/	/	12.9			1.3			54.9			6.6			Xia [2]
Excavator	/	/	7.9			6			42.6			0.3			Fu [24]
Excavator	/	/	11.7			3.3			31.1			1.4			Frey [26]

**Table 3:** The emission reduction ratio of different emission reduction techniques for testing construction machinery.

No.	Emission Reduction Techniques	CO	THC	NO_X_	PM	Experimental Conditions	Source
1	Enhanced model 3DOC + CDPF	49.5%	89.8%	/	99.5%	Actual operating conditions	This study
2	Burner + DOC + CDPF	91.7%	95.1%	/	99.4%		
3	DOC + CDPF + Copper-based molecular sieves SCR + ASC	92.8%	90.9%	80.5%	97.5%		
4	DOC + CDPF + Vanadium based SCR + ASC	79.5%	85.5%	67.3%	87.2%		
5	DOC + CDPF + Vanadium based SCR + ASC	94.8%	92.7%	60.3%	99.3%		
6	Clean fuel + lubricant additives	12.8%	/	/	72%		
7	DOC + CDPF + Copper-based molecular sieves SCR + ASC	41.6%	/	70.6%	98.9%		
8	DOC + CDPF + Copper-based molecular sieves SCR + ASC	78.2%	/	76.5%	96.6%		
9	DOC + CDPF + SCR	97.6%	98.1%	90%	98%	NRSC	Hu [16]
10	DOC + CDPF + SCR	55%	57.1%	57.7%	95%	NRTC	Hu [16]
11	EGR + DOC + DPF	Nearly 100%	Nearly 100%	24.3%	/	NRTC	Hu [29]
12	DOC + DPF	85%	50–80%	/	90%	Double idle + free acceleration	Shao [30]
13	DOC + CDPF	/	/	85%	90.9%	NRTC	Zhang [31]
14	DOC + SCR	/	/	90%	/	NRTC	Ummel [32]
15	DOC + DPF + SCR	97%	86%	70%	80%	NRSC	Sun [33]

The emission reduction ratio of the machines No. 6, 7, and 8 was calculated based on the unprocessed raw data collected by the equipment.

## Data Availability

Not applicable.
